# Response surface optimization of enzymatic hydrolysis and ROS scavenging activity of silk sericin hydrolysates

**DOI:** 10.1080/13880209.2022.2032208

**Published:** 2022-02-11

**Authors:** Keerati Joyjamras, Chatchai Chaotham, Pithi Chanvorachote

**Affiliations:** a Graduate Program of Pharmaceutical Sciences and Technology, Faculty of Pharmaceutical Sciences, Chulalongkorn University, Bangkok, Thailand; b Department of Biochemistry and Microbiology, Faculty of Pharmaceutical Sciences, Chulalongkorn University, Bangkok, Thailand; c Department of Pharmacology and Physiology, Faculty of Pharmaceutical Sciences, Chulalongkorn University, Bangkok, Thailand; d Center of Excellence in Cancer Cell and Molecular Biology, Faculty of Pharmaceutical Sciences, Chulalongkorn University, Bangkok, Thailand

**Keywords:** Waste product, RSM, Alcalase^®^, antioxidant

## Abstract

**Context:**

Sericin, a protein found in wastewater from the silk industry, was shown to contain a variety of biological activities, including antioxidant. The enzymatic conditions have been continuously modified to improve antioxidant effect and scavenging capacity against various free radicals of silk sericin protein.

**Objective:**

Variables in enzymatic reactions, including pH, temperature and enzyme/substrate ratio were analysed to discover the optimum conditions for antioxidant activity of sericin hydrolysates.

**Materials and methods:**

Hydrolysis reaction catalysed by Alcalase^®^ was optimized through response surface methodology (RSM) in order to generate sericin hydrolysates possessing potency for % inhibition on 2,2-diphenyl-1-picrylhydrazyl (DPPH) radicals, ferric-reducing power and peroxyl scavenging capacity. Flow cytometry was performed to evaluate cellular ROS level in human HaCaT keratinocytes and melanin-generating MNT1 cells pre-treated either with 20 mg/mL RSM-optimized sericin hydrolysates or 5 mM *N*-acetyl cysteine (NAC) for 60 min prior exposure with 1 mM hydrogen peroxide (H_2_O_2_).

**Results:**

Among these three variables, response surface plots demonstrate the major role of temperature on scavenging capacity of sericin hydrolysates. Sericin hydrolysates prepared by using Alcalase^®^ at RSM-optimized condition (enzyme/substrate ratio: 1.5, pH: 7.5, temperature: 70 °C) possessed % inhibition against H_2_O_2_ at 99.11 ± 0.54% and 73.25 ± 8.32% in HaCaT and MNT1 cells, respectively, while pre-treatment with NAC indicated the % inhibition only at 30.26 ± 7.62% in HaCaT and 51.05 ± 7.14% in MNT1 cells.

**Discussion and conclusions:**

The acquired RSM information would be of benefit for further developing antioxidant peptide from diverse resources, especially the recycling of waste products from silk industry.

## Introduction

The rapid expansion of industries to supply consumable products globally unavoidably causes ecological problems (Zhu et al. 2019). Without proper management, sericin protein present in the degumming water used in silk processing results in a high level of chemical oxygen demand (COD), which contributes to water pollution (Pakdel et al. 2016). In seeking to recycle the wastewater from silk production, several researchers have discovered the potential benefits of silk sericin (Kunz et al. 2016; Cao and Zhang 2017; Liu et al. 2020). Silk protein, which is produced from *Bombyx mori* Linnaeus (Bombycidae) comprises 25–30% sericin protein wrapped around fibroin fibre (Jena et al. 2018). The globular structure of water-soluble sericin consists of diverse amino acids, among which serine, histidine, glycine, threonine, tyrosine, aspartate and glutamine are predominant (Kunz et al. 2016). Recently, several biological functions of sericin have been reported, including antioxidant activity (Ersel et al. 2016; Ampawong et al. 2017; Manesa et al. 2020).

Scavenging activity, or the capability to eliminate the unpaired electron in oxygen and other molecules, is one of the major characteristics of antioxidant compounds (Shahidi and Zhong 2015). Through direct interaction with reactive oxygen species (ROS), antioxidants can restrain oxidative stress and prevent propagation of oxidative chain reactions, which would otherwise damage cellular organelles (He et al. 2017). Moreover, the application of natural antioxidants has also been researched in food, pharmaceutical and cosmetic products (Obrenovich et al. 2011; Ribeiro et al. 2015). It is widely accepted that the antioxidant capacities of natural compounds can be accessed through various *in vitro* assays, including 2,2-diphenyl-1-picrylhydrazyl (DPPH) radical-scavenging activity, ferric-reducing antioxidant power (FRAP) and oxygen radical absorbance capacity (ORAC) (Gulcin 2020). Based on the donation of a single electron to free radicals and ferric ions (Fe^3+^), antioxidant activity can be respectively determined using DPPH and FRAP assays (Apak et al. 2016). Despite their simplicity and repeatability, DPPH and FRAP assays carry the drawback of irrelevance to biological ROS and physiological conditions (Ndhlala et al. 2010). Therefore, ORAC assay, which generates peroxyl radicals (ROO**˙**), is introduced to examine the translocation of hydrogen atoms from antioxidant to oxygen molecules (Huang et al. 2005). According to diverse mechanisms of action, ROS scavenging activity of antioxidant compound is recommended to evaluated through several methods (Ou et al. 2002; Alam et al. 2013; Dienaitė et al. 2019).

Intriguingly, the ROS scavenging capacity of peptides, in both their natural and hydrolysed forms, is well established (Wang et al. 2015; Jiang et al. 2017; Zhang et al. 2019). While the antioxidant potential of sericin and sericin hydrolysates has largely been evidenced using DPPH assay (Manosroi et al. 2010; Jena et al. 2018; Miguel and Álvarez-López 2020), the study of the scavenging activity of hydrolysed sericin prepared by specific enzyme against diverse types of free radicals is still limited (Fan et al. 2010; Takechi et al. 2014). To optimize conditions in both laboratory and industrial scenarios, response surface methodology (RSM), a type of statistical and mathematical analysis, has been broadly applied (Vázquez et al. 2017). RSM gathers the effects of different independent factors to generate an applicable model for desired output (Yolmeh and Jafari 2017). Variables in enzymatic reactions, including pH, temperature and enzyme/substrate ratio were acquired from RSM in this study and analysed to discover the optimum conditions for antioxidant activities of sericin hydrolysates in DPPH, FRAP and ORAC assays. The obtained information would be of benefit for recycling and utilizing sericin, a waste product from the silk industry, as a potent antioxidant compound.

## Material and methods

### Materials

Lyophilized silk sericin powder was kindly provided by Ruenmai-baimon, LTD., Surin Province, Thailand. Porcine pancreas trypsin (EC 3.4.21.4), papain (EC 3.4.22.2), Alcalase^®^ (EC 3.4.21.62), 2,2-diphenyl-1-picrylhydrazyl (DPPH), 2,4,6-tri(2-pyridyl)-s-triazine (TPTZ), ferric chloride hexahydrate (FeCl_3_⋅6H_2_O), 37% hydrochloric acid (HCl) solution, 1 M sodium hydroxide (NaOH) solution, fluorescein, 2,2′-azobis-2-methyl-propanimidamide dihydrochloride (AAPH), sodium dodecyl sulfate (SDS), Coomassie brilliant blue R-250, isopropanol, ethanol, acetic acid solution, 2′,7′-dichlorofluorescein diacetate (DCFH_2_-DA) and *N*-acetyl cysteine (NAC) were purchased from Sigma-Aldrich (St. Louis, MO, USA). A bicinchoninic acid (BCA) protein assay kit used for determination of total protein content was procured from Thermo Scientific (Rockford, IL, USA). MilliporeSigma (Burlington, MA, USA) was the source of 3% w/w hydrogen peroxide (H_2_O_2_) solution.

### Enzymatic hydrolysis of sericin

Three commercial proteases, including trypsin, papain and Alcalase^®^ were chosen to hydrolyse sericin at the optimum conditions recommended by the manufacturers as indicated in Table 1. Briefly, sericin powder was dispersed in de-ionized water at a concentration of 2% w/v. The protein suspension was heated at 95 °C for 10 min until complete solubilization before immediate cool down on ice to room temperature. The pH of sericin solution was adjusted by adding 0.1 M NaOH to the desired condition depending on used proteases, and then, hydrolysis experiments were carried out in a 50-mL vessel. To stop the enzymatic reaction, the solution was heated at 100 °C for 5 min and quickly chilled on ice to room temperature. Sericin hydrolysates in the supernatant were collected after centrifugation at 3,500 *g* for 30 min at 4 °C before being subjected to freeze-drying. The hydrolysed sericin powder was stored at −20 °C until use in further experiments.

**Table 1.** Optimum enzymatic condition following manufacturer’s instructions.

Protease enzyme	Factors			
	pH	E/S (w/w)	Temperature (°C)	Time (h)
Alcalase^®^	8	2	60	3
Papain	7	2	60	3
Trypsin	8	2	40	3

E/S: Enzyme/Substrate ratio.

### DPPH radical scavenging activity

DPPH radical scavenging activity of sericin hydrolysates was determined according to the method of Agrawal et al. (2016). Briefly, 100 μL of sericin hydrolysates (4 mg/mL in de-ionized water) was mixed with 100 μL of DPPH solution (0.1 mM in 95% ethanol) in a 96-well plate and incubated at room temperature for 60 min in the dark. The absorbance intensity (Abs) of DPPH radicals was determined using a microplate reader (Anthros, Durham, NC, USA) at 517 nm. The % inhibition of DPPH was calculated as follows:

(1) $$\text{DPPH}\;\text{scavenging}\;\text{inhibition}\;(\%)\;=\;\frac{\text{Abs}(\text{Control})-\text{Abs}(\text{Sample})}{\text{Abs}(\text{Control})}\text{ × }100$$

### Ferric-reducing antioxidant power (FRAP) assay

FRAP assay was performed to evaluate the ferric-reducing antioxidant power of sericin hydrolysates (e Silva et al. 2017). Sericin hydrolysates at 4 mg/mL in de-ionized water (50 μL) were allowed to react with 150 μL of 0.3 M FRAP reagent in acetate buffer, pH 3.6 (10 mM 2,4,6‐tripyridyl‐*S*‐triazine: 40 mM HCl: 20 mM FeCl_3_⋅6H_2_O at 10:1:1 ratio). The reaction mixture was kept from light at room temperature for 15 min, and then, the absorbance of ferrous ion (Fe^2+^) complex was examined using a microplate reader (Anthros, Durham, NC, USA) at 595 nm. A calibration curve of Fe^2+^ was used to calculate the reducing power, which was presented as Fe^2+^ equivalence (eq.)/mg of sericin hydrolysates.

### Oxygen radical absorbance capacity (ORAC) assay

ORAC assay was performed in 75 mM phosphate buffer (pH 7.4). Briefly, the mixture of 25 μL sericin hydrolysates (4 mg/mL in PBS) and fluorescein solution at final concentration of 70 nM (150 μL) in a 96-well clear bottom black plate was preincubated at 37 °C for 15 min. Subsequently, 25 μL of AAPH was rapidly added to the mixture to get the final concentration of 12 mM. The plate was shaken for 5 s before measurement of the fluorescence intensity of fluorescein using a CLARIOstar plus microplate reader (BMG LABTECH, Ortenberg, Germany) with excitation wavelength at 485 nm and emission wavelength at 520 nm every 90 s for 150 min. Instead of the testing solution, phosphate buffer solution (PBS) at pH 7.4 was chosen as a blank, while Trolox was selected as a calibration solution. Fluorescence measurements were normalized to the curve of the PBS blank. The area under the fluorescence decay curve (AUC) was calculated as follows:

$$\text{AUC}=1+\sum_{i=150}^{i=1.5}f_{i}/f_{0}$$

(2) $$\text{net}\;\text{AUC}={\text{AUC}}_{\text{antioxidant}}-{\text{AUC}}_{\text{blank}}$$

where f_0_ is the initial fluorescence reading at 0 min, f_i_ is the fluorescence reading at time i min

The regression equation between the net AUC and the Trolox concentration was calculated. ORAC values were expressed as μmol Trolox equivalence (TE)/mg of sericin hydrolysates (e Silva et al. 2017).

### Response surface methodology for optimization of enzymatic hydrolysis conditions

The three independent variables of pH, enzyme/substrate ratio and temperature at three levels were generated in a Box–Behnken design using a trial version of Design-Expert^®^ 11 software (Stat-Ease Inc., Minneapolis, MN, USA). The levels and range of each variable are indicated in Table 2. The response data obtained from each designed condition were determined by the following quadratic polynomial equation:

(3) $$Y\;=\;\beta_{0}+\beta_{1}x_{1}+\beta_{2}x_{2}+\beta_{3}x_{3}+\beta_{11}x_{1}^{2}+\beta_{22}x_{2}^{2}+\beta_{33}x_{3}^{2}+\beta_{12}x_{1}x_{2}+\beta_{13}x_{1}x_{3}+\beta_{23}x_{2}x_{3}$$

where *Y* is the response variable (DPPH, FRAP or ORAC); *β_0_* is an offset constant; *β_1_, β_2_,* and *β_3_* are linear regression coefficients; *β_11_, β_22_ and β_33_* are quadratic effects; *β_12_, β_13_* and *β_23_* represent interaction effects; *x_1_, x_2_* and *x_3_* represent independent variables in this model.

**Table 2.** Independent variables and their levels in Box–Behnken design.

Variables	Code	Level and range		
		−1	0	1
pH	A	7	8	9
E/S (w/w)	B	1	2	3
Temperature (°C)	C	50	60	70

E/S: Enzyme/Substrate ratio.

The analysis of variance (ANOVA) was performed using RSM software Minitab.16 to determine the adequacy of models through lack of fit value, coefficient determination (R^2^) and adjusted-R^2^ (Mang et al. 2015). Statistical significance was considered at *p* < 0.05.

### Molecular weight distribution of sericin hydrolysates

Constituents of hydrolysed sericin were analysed via sodium dodecyl sulphate-polyacrylamide gel electrophoresis (SDS-PAGE). Equal amounts of protein mixed with loading dye were heated at 95 °C for 5 min and added onto 12% (w/v) gel of SDS-PAGE. The separated protein constituents were stained overnight with Coomassie brilliant blue R-250 solution. The molecular weight distribution of hydrolysed sericin was clearly observed after destaining the gel with isopropanol: acetic acid: water (10%: 10%: 80% v/v) solution (Laemmli and Favre 1973). Additionally, the size distribution profile of RSM-optimized sericin hydrolysates was also generated through fast protein liquid chromatography (FPLC) coupled with HiPrep 16/60 Sephacryl S-200 HR column (GE Healthcare, Stockholm, Sweden). Briefly, sericin hydrolysates at 4 mg/mL was prepared in Tris-HCl buffer (50 mM Tris–HCl, pH 8.0, 200 mM NaCl) for loading on the size exclusion chromatography column preequilibrated with Tris-HCl buffer. Then, the protein sample was eluted with Tris-HCl buffer at a flow rate of 1 mL/min. Absorbance of the eluent at 214 nm was determined to estimate protein concentration.

### Cell culture

Human keratinocytes (HaCaT) and human melanoma MNT1 cells were obtained from the American Type Culture Collection (ATCC, Manassas, VA, USA). Human keratinocytes were cultured in Dulbecco’s Modified Eagle Medium (DMEM), supplemented with 2 mmol/L l-glutamine, 10% (v/v) fetal bovine serum (FBS) and 100 units/mL of penicillin/streptomycin (Gibco, Gaithersburg, MD, USA). Meanwhile, melanin-generating MNT1 cells were cultured in DMEM supplemented with 20% FBS, 10% AIM-V medium (Gibco, Gaithersburg, MD, USA), 2 mmol/L l-glutamine and 100 units/mL of penicillin/streptomycin. Cells that reached 70-80% confluence under 5% CO_2_ at 37 °C were used in further experiments.

### Determination of cellular ROS level via flow cytometry

Cells seeded at a density of 1 × 10^5^ cells/well in six-well plates were incubated with 10 μM DCFH_2_-DA for 30 min at 4 °C while kept from light. Then, the cells were washed with PBS and pre-treated either with 5 mM NAC, 20 mg/mL sericin hydrolysates or 20 mg/mL unhydrolysed sericin for 60 min prior to exposure to 1 mM H_2_O_2_. After 30 min of treatment with H_2_O_2_, the cells were resuspended in PBS and immediately subjected to flow cytometry using Guava easyCyte benchtop flow cytometers (EMD Millipore, Darmstadt, Germany) for measurement of cellular fluorescence intensity of DCF at excitation/emission wavelengths of 488/538 nm. Cellular ROS level was a relative value of mean fluorescence intensity quantified by Guava InCyte version 3.1 software (EMD Millipore) between specific treatment and untreated control cells.

### Statistical analysis

All experimental data were presented as means ± standard error of the mean (SEM). SPSS version 22 (IBM Corp., Armonk, NY, USA) with one-way analysis of variance (ANOVA) followed by Tukey’s *post hoc* test was performed for the statistical analysis. Any *p*-value under 0.05 was considered as statistical significance.

## Results

### ROS scavenging activity of sericin hydrolysates prepared from various protease enzymes

**Figure 1.**
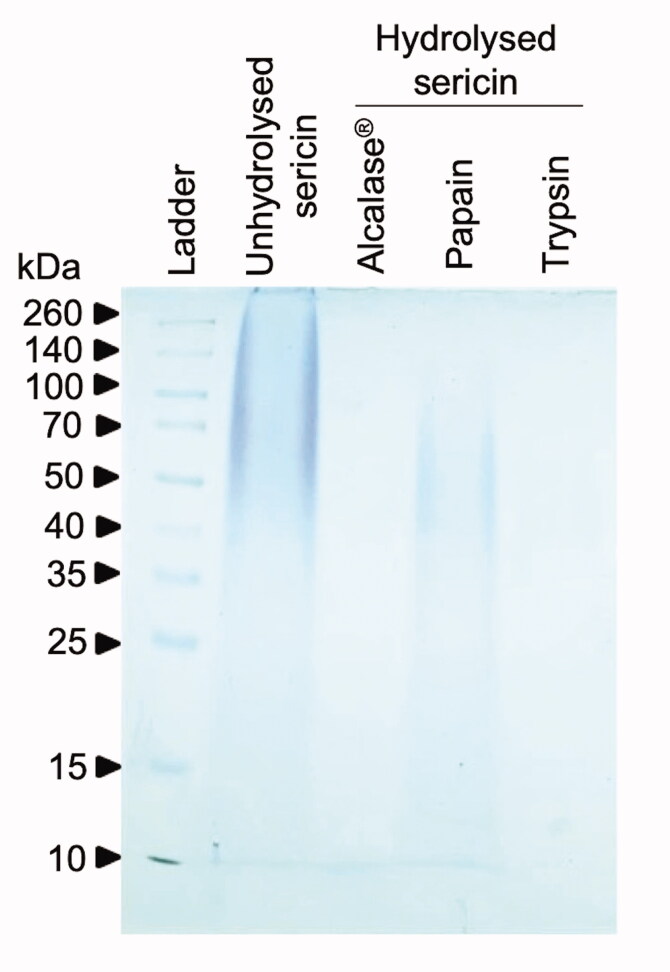
Distribution of protein composition in sericin hydrolysates prepared from different commercial enzymes in SDS-PAGE analysis.

Initially, silk sericin was digested by three commercial proteases to identify the hydrolysed sericin that possessed the highest antioxidant activity. After 3 h of enzymatic reaction following the manufactures’ conditions, SDS-PAGE analysis revealed the alteration of protein constituents in sericin hydrolysates (Figure 1). The absence of high molecular weight (∼100–260 kDa) proteins indicated the enzymatic function of Alcalase^®^, papain and trypsin in such conditions. Only protein at ∼10 kDa was presented in sericin hydrolysates obtained from Alcalase^®^ while papain hydrolysed-sericin consisted with proteins ranging from ∼10 to 100 kDa. It should be noted that staining with Coomassie brilliant blue R-250 barely detected protein components in sericin hydrolysates derived from trypsin reaction. Antioxidant activity of the sericin hydrolysates prepared from these three commercial enzymes was then assessed through DPPH, FRAP and ORAC assays. The greater scavenging activity against DPPH and ROO**˙** radials as respectively indicated by greater % DPPH inhibition and ORAC values was noted in all hydrolysed sericins compared with unmodified sericin (Table 3). Interestingly, only sericin hydrolysates obtained from Alcalase^®^ achieved better ferric-reducing power, as evidenced by its higher FRAP value when compared with unhydrolyzed sericin. It is worth noting that modification with papain and trypsin decreased ferric-reducing power of sericin proteins. Sericin hydrolysates obtained from Alcalase^®^ demonstrated the highest antioxidant capacities in all ROS scavenging assays (% DPPH inhibition = 19.71 ± 0.13%, FRAP activity = 435.50 ± 10.13 µmol Fe^2+^ eq./mg protein and ORAC value = 4,383.92 ± 12.23 µmol TE/mg protein). Among the three commercial enzymes, the lowest ROS scavenging activities were observed in sericin hydrolysates obtained by using papain. In summary, Alcalase^®^ was selected as the best candidate protease for further optimization of antioxidant activity of sericin hydrolysates.

**Table 3.** Antioxidant activity of hydrolysed silk sericin from various proteases.

Protease Enzyme	Factors				Response variable		
	pH	E/S (w/w)	Temperature (°C)	Time (h)	DPPH^a^ (% Inhibition)	FRAP value^a^ (µmol Fe^2+^ eq./mg protein)	ORAC value^a^ (µmol TE/mg protein)
- (Unhydrolysed sericin)	–	–	–	–	9.43 ± 0.10	327.50 ± 11.87	2,767.72 ± 30.97
Alcalase^®^	8	2	60	3	19.71 ± 0.13	435.50 ± 10.13	4,383.92 ± 12.23
Papain	7	2	60	3	12.64 ± 0.16	274.68 ± 10.47	3,897.33 ± 23.64
Trypsin	8	2	40	3	14.43 ± 0.09	284.51 ± 6.46	4,026.85 ± 20.17

E/S: Enzyme/Substrate ratio, TE: Trolox equivalence.

^a^Obtained from three independent experiments.

### Response surface optimization of enzymatic reaction for sericin hydrolysates prepared by using Alcalase^®^

To investigate the influence of enzymatic conditions on the antioxidant activity of sericin hydrolysates, the independent factors, pH, enzyme/substrate ratio and temperature, were resolved by RSM. The experimental conditions and resultant antioxidant activities generated through Box-Behnken design of RSM are shown in Table 4. From the 17 experimental conditions, the antioxidant responses of sericin hydrolysates ranged as follows: % inhibition of DPPH: 11.21-20.37%, FRAP: 362.03-455.93 µmol Fe^2+^ eq/mg protein and ORAC: 3,568.68-4,597.03 µmol TE/mg protein.

**Table 4.** Box–Behnken factorial design of enzymatic hydrolysis and antioxidant response.

Run	Independent variables			Responses		
	A: pH	B: E/S (w/w)	C: Temperature (°C)	Y_1_: DPPH (% Inhibition)	Y_2_: FRAP (µmol Fe^2+^ eq./mg sample)	Y_3_: ORAC (µmol TE/mg sample)
1	9	2	70	14.67	455.93	4,591.43
2	7	2	70	20.37	449.07	4,504.01
3	8	3	70	18.89	422.03	4,156.45
4	8	2	60	14.83	439.90	4,086.49
5	8	1	50	12.86	364.41	3,617.85
6	8	2	60	14.69	433.76	4,073.43
7	9	3	60	11.21	383.05	4,052.36
8	8	3	50	14.61	404.24	3,568.68
9	9	2	50	13.37	376.95	3,805.73
10	7	3	60	16.87	414.41	3,908.42
11	8	2	60	14.71	443.90	4,086.49
12	7	1	60	13.58	362.03	4,142.16
13	8	2	60	14.15	442.04	4,097.94
14	9	1	60	14.20	445.76	4,283.99
15	7	2	50	12.86	368.64	3,696.03
16	8	1	70	18.31	452.54	4,597.03
17	8	2	60	14.37	449.32	4,064.59

E/S: Enzyme/Substrate ratio, TE: Trolox equivalence.

Data were analysed using model regression analysis with *p* < 0.05 using Design-Expert^®^ 11 software. Polynomial equations were as follows:

(4) $$Y1=14.5508-1.2788A\;+0.3292B+2.3169C-1.5689AB-1.5545AC-0.2932\text{BC }-\;0.7172A^{2}+0.1325B^{2}+1.4844C^{2}$$

(5) $$Y2\;=441.784\;+8.4431A\;-\;0.1266B\;+\;33.1675C\;-\;28.7722\text{AB }-\;0.3609\text{AC }-17.5847\text{BC }-\;19.3137A^{2}-21.1577B^{2}-9.8212C^{2}$$

(6) $$Y3\;=\;4081.7867+60.3617A\;-\;119.3904B\;+\;395.0788C\;+\;0.5267\text{AB }-5.5683\text{AC }-\;97.8541\text{BC }+\;89.6237A^{2}-74.6771B^{2}-22.1088C^{2}$$

where Y1 is the response from DPPH, Y2 is the response from FRAP, Y3 is the response from ORAC, A is pH, B is enzyme/substrate ratio and C is temperature.

Following statistical analysis of the quadratic model of DPPH (Table 5), FRAP (Table 6) and ORAC responses (Table 7), the significance of all models was evidenced with *p* < 0.05. Additionally, both R^2^ and adjusted R^2^ ranging between 0.9143 and 0.9988 as well as the non-significance (*p* > 0.05) of lack of fit indicated the high accuracy of predicted responses from these quadratic models (Ravikumar et al. 2006; Mushtaq et al. 2015). Notably, the predicted R^2^ is close to 1 in the quadratic model of response of DPPH (0.8829) and ORAC (0.9870) assays, as presented in Tables 6 and 8, respectively, while the predicted R^2^ value is about 0.4991 for FRAP response (Table 7). The positive linear effects of pH (A) and temperature (C) were shown to be significant for scavenging activity determined by DDPH, FRAP and ORAC assays. However, the enzyme/substrate ratio (B) was shown to be clearly positive for DDPH and ORAC, but not for FRAP assay. Or to put it conversely, the quadratic effect of the enzyme/substrate ratio (B^2^) only significantly affected ORAC and FRAP responses, while all ROS scavenging activities were found to be modulated by the quadratic effects of pH (A^2^) and temperature (C^2^).

**Table 5.** ANOVA for quadratic model of DPPH response.

Source	Sum of Squares	df	Mean Square	F-value	*p*-value
Model	87.88	9	9.76	72.96	<0.0001*
pH (A)	13.08	1	13.08	97.75	<0.0001*
E/S (B)	0.8671	1	0.8671	6.48	0.0384*
Temperature (C)	42.94	1	42.94	320.87	<0.0001*
AB	9.85	1	9.85	73.57	<0.0001*
AC	9.67	1	9.67	72.23	<0.0001*
BC	0.3439	1	0.3439	2.57	0.1530
A²	2.17	1	2.17	16.19	0.0050*
B²	0.0740	1	0.0740	0.5526	0.4814
C²	9.28	1	9.28	69.32	<0.0001*
Residual	0.9368	7	0.1338		
Lack of Fit	0.6191	3	0.2064	2.60	0.1895
Pure Error	0.3177	4	0.0794		
Cor Total	88.82	16			
R²	0.9895				
Adjusted R²	0.9759				
Predicted R²	0.8829				
Adeq Precision	32.1496				
C.V.%	2.44				

E/S: Enzyme/Substrate ratio, *p < 0.05.

**Table 6.** ANOVA for quadratic model of FRAP response.

Source	Sum of Squares	df	Mean Square	F-value	*p*-value
Model	18183.51	9	2020.39	19.97	0.0003*
pH (A)	570.29	1	570.29	5.64	0.0493*
E/S (B)	0.1283	1	0.1283	0.0013	0.9726
Temperature (C)	8800.69	1	8800.69	87.00	<0.0001*
AB	3311.35	1	3311.35	32.74	0.0007*
AC	0.5211	1	0.5211	0.0052	0.9448
BC	1236.89	1	1236.89	12.23	0.0100*
A²	1570.61	1	1570.61	15.53	0.0056*
B²	1884.83	1	1884.83	18.63	0.0035*
C²	406.13	1	406.13	4.01	0.0851
Residual	708.09	7	101.16		
Lack of Fit	578.82	3	192.94	5.97	0.0585
Pure Error	129.27	4	32.32		
Cor Total	18891.59	16			
R²	0.9625				
Adjusted R²	0.9143				
Predicted R²	0.4991				
Adeq Precision	13.1588				
C.V.%	2.41				

E/S: Enzyme/Substrate ratio, *p < 0.05.

**Table 7.** ANOVA for quadratic model of ORAC response.

Source	Sum of Squares	df	Mean Square	F-value	*p*-value
Model	1.48 × 10^6^	9	1.65 × 10^5^	636.50	<0.0001*
pH (A)	29148.25	1	29148.25	112.31	<0.0001*
E/S (B)	1.14 × 10^5^	1	1.14 × 10^5^	439.39	<0.0001*
Temperature (C)	1.24 × 10^6^	1	1.24 × 10^6^	4811.45	<0.0001*
AB	1.11	1	1.11	0.0043	0.9497
AC	124.03	1	124.03	0.4779	0.5116
BC	38301.75	1	38301.75	147.58	<0.0001*
A²	33820.70	1	33820.70	130.32	<0.0001*
B²	23480.70	1	23480.70	90.48	<0.0001*
C²	2058.09	1	2058.09	7.93	0.0259*
Residual	1816.69	7	259.53		
Lack of Fit	1146.02	3	382.01	2.28	0.2215
Pure Error	670.67	4	167.67		
Cor Total	1.48 × 10^6^	16			
R²	0.9988				
Adjusted R²	0.9972				
Predicted R²	0.9870				
Adeq Precision	83.4263				
C.V.%	0.40				

E/S: Enzyme/Substrate ratio, *p < 0.05.

**Table 8.** Antioxidant activity of sericin hydrolysed by Alcalase^®^ under RSM-optimized condition.

Optimized condition	Response	Predicted value	Observed value^a^	% Error
pH: 7.5, E/S: 1.5, Temperature: 70ºC Time = 3 h	DPPH (% Inhibition)	19.14	18.65 ± 0.21	2.56
	FRAP (µmol Fe^2+^ eq./mg sample)	455.93	462.31 ± 10.82	1.40
	ORAC (µmol TE/mg sample)	4,532.23	3,811.00 ± 39.37	15.91

E/S: Enzyme/Substrate ratio, TE: Trolox equivalence.

^a^Obtained from three independent experiments.

Table 5 also indicates the interactive effect of two variables on scavenging activity against DPPH radicals. The interaction between pH and enzyme/substrate ratio (AB) and between pH and temperature (AC) clearly affected % inhibition of DPPH of sericin hydrolysates prepared from Alcalase^®^. Similarly, the significant effects on FRAP antioxidant response arose from pH and enzyme/substrate ratio (AB) interaction as well as enzyme/substrate ratio and temperature (BC) interaction (Table 6). Surprisingly, only the interaction between enzyme/substrate ratio and temperature (BC) was significantly positive on scavenging activity against ROO**˙** (Table 7). Taken together, temperature seems to have the greatest influence on ROS scavenging activity of sericin hydrolysates determined by DPPH, FRAP and ORAC assays, as evidenced in multiple linear regression analysis of linear, quadratic and interactive effects.

**Figure 2.**
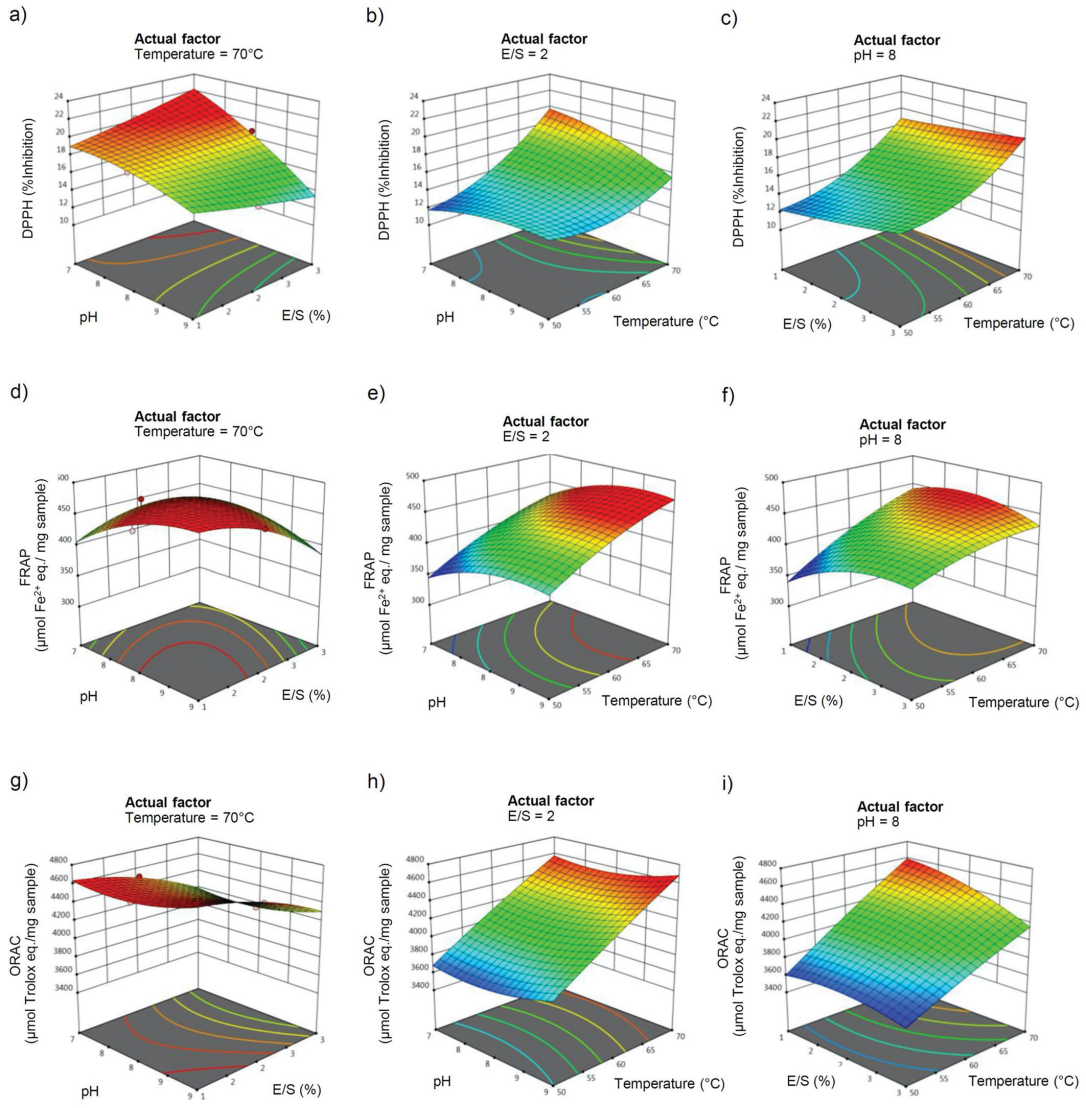
Response surface plots depicting the effects of pH, enzyme/substrate ratio (E/S) and temperature on antioxidant activity of sericin hydrolysates prepared by using Alcalase^®^ against (a–c) DPPH free radicals, (d–f) ferric ions (Fe^3+^) and (g–i) peroxyl radicals.

The effect of correlative adjustment of two variables involved in enzymatic reactions on antioxidant activity of sericin hydrolysates prepared by using Alcalase^®^ in DPPH, FRAP and ORAC assays is shown in response surface three-dimension graphs (Figure 2). Correspondence with the regression analysis of interactive effect, the major influence of temperature (∼70 °C) during enzymatic process of Alcalase^®^ on all ROS scavenging activities is obviously demonstrated in the correlative alteration with both pH (Figure 2(b, e and h)) and enzyme/substrate ratio (Figure 2(c, f and i)). The response surface plots also demonstrate that pH variations combined with variations in enzyme/substrate ratio alter only the % inhibition of DPPH (Figure 2(a)), but not ferric-reducing power (Figure 2(d)) or oxygen radical absorbance capacity (Figure 2(g)). Meanwhile, the correlative adjustment of enzyme/substrate ratio with other variables plays a minor role in the modulation of all ROS scavenging capacities.

### ROS scavenging activity of sericin hydrolysates modified by using Alcalase^®^ under RSM-optimized conditions

Based on response surface analysis, the optimum conditions for preparation of sericin hydrolysates with maximized antioxidant activities in DPPH, FRAP and ORAC assays was generated through numerical optimization in Design-Expert^®^ 11 software. After 3 h of enzymatic reaction performed according to RSM-optimized conditions at pH 7.5, enzyme/substrate ratio of 1.5 (w/w) and temperature of 70 °C, sericin hydrolysates were evaluated for ROS scavenging activity. As presented in Table 8, sericin hydrolysates derived after the reaction of Alcalase^®^ under the RSM-optimized condition possessed antioxidant activities, including % inhibition of DPPH, ferric-reducing power and oxygen radical absorbance capacity close to predicted values. It is noted that the lower variation between predicted and observed responses in DPPH and FRAP assays is indicated by lower % error range (between 1.46 and 2.50), compared with the 15.91% error in ORAC response.

### Sericin hydrolysates ameliorate H_2_O_2_-induced oxidative stress in human keratinocytes and melanin-generating cells

**Figure 3.**
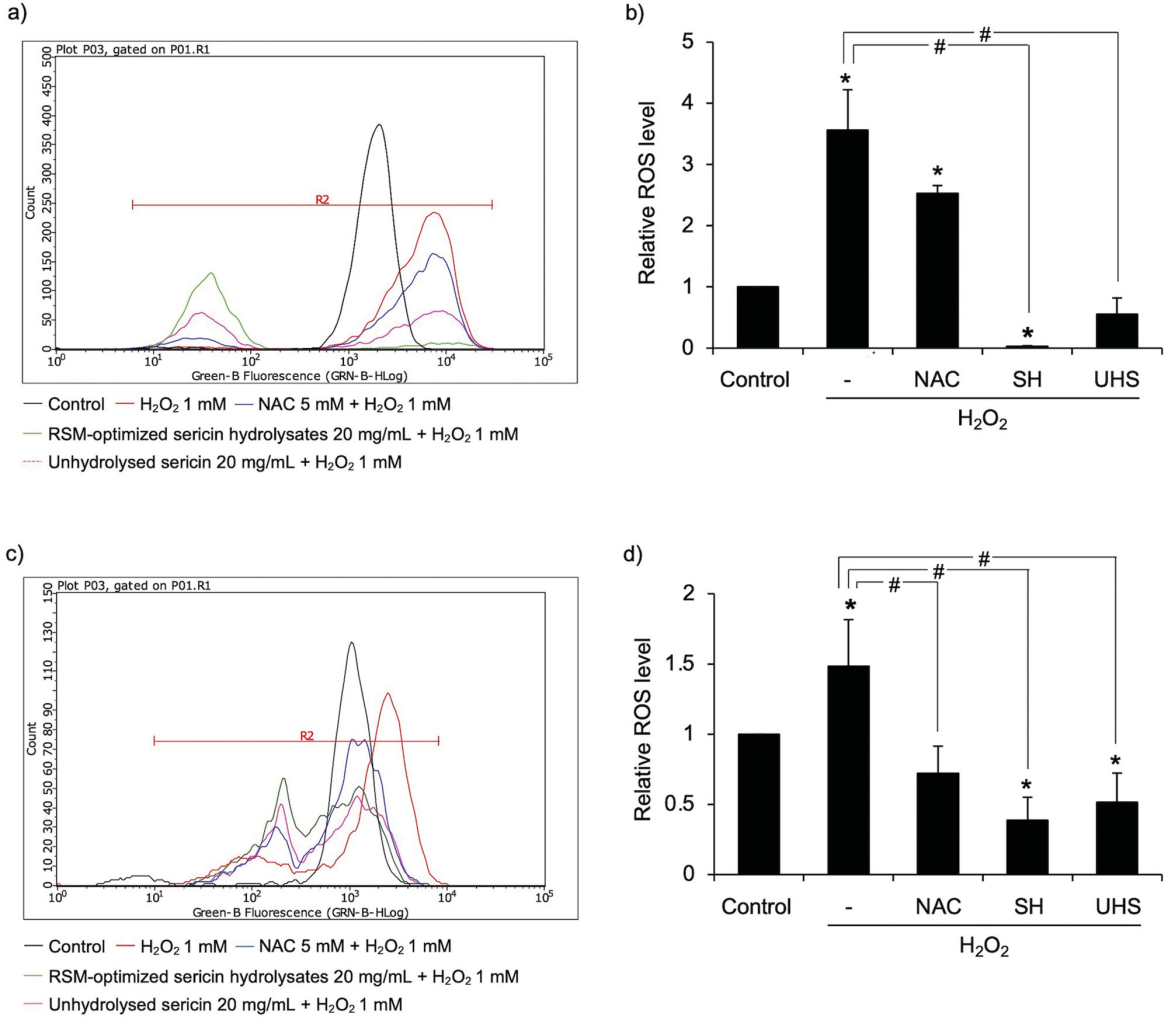
Cellular antioxidant activity of sericin hydrolysates prepared from Alcalase^®^ under RSM-optimized conditions. The alteration of cellular ROS levels is presented in flow cytometry histograms of (a) human keratinocyte HaCaT and (c) human melanin-generating MNT1 cells stained with DCFH_2_-DA fluorescence probe. Preculture with 5 mM N-acetyl cysteine (NAC), 20 mg/mL unhydrolysed sericin (UHS) or 20 mg/mL RSM-optimized sericin hydrolysates (SH) obviously diminished the relative ROS levels in (b) keratinocytes and (d) MNT1 cells after exposure to 1 mM hydrogen peroxide (H_2_O_2_) for 30 min. Data are presented as means ± SEM from three independent experiments. *p < 0.05 compared with untreated control cells. ^#^*p* < 0.05 compared with the cells treated only with H_2_O_2_.

The ROS scavenging activity of sericin hydrolysates derived from Alcalase^®^ was further evaluated in cell-based assay. Because the potential benefits of sericin are widely recognized in cosmeceuticals (Kunz et al. 2016), the antioxidant activity of sericin hydrolysates obtained from Alcalase^®^ was investigated in skin epidermal cells, including human keratinocytes and melanin-generating cells. Flow cytometry histograms illustrate the augmented cellular ROS detected by DCFH_2_-DA fluorescence probe in keratinocytes (Figure 3(a)) and melanocytes (Figure 3(c)) after exposure to 1 mM H_2_O_2_ for 30 min. Intriguingly, preculture with 20 mg/mL of Alcalase^®^ sericin hydrolysates for 1 h dramatically reversed cellular oxidative stress induced by H_2_O_2_. The lower relative ROS levels were indicated in the cells preincubated with sericin hydrolysates compared with the pre-treatment either with unhydrolysed sericin (20 mg/mL) or 5 mM NAC, a well-known antioxidant (Figure 3(b and d)). It is worth noting that RSM-optimized sericin hydrolysates possess greater % inhibition against H_2_O_2_ in both HaCaT (99.11 ± 0.54%) and MNT1 cells (73.25% ± 8.32%) compared with unhydrolysed sericin (HaCaT: 88.52 ± 2.43%, MNT1:64.99 ± 7.83%) or NAC (HaCaT: 30.26 ± 7.62%, MNT1:51.05 ± 7.14%). These results confirm the antioxidant potential of sericin hydrolysates prepared by using Alcalase^®^ under RSM-optimized conditions.

**Figure 4.**
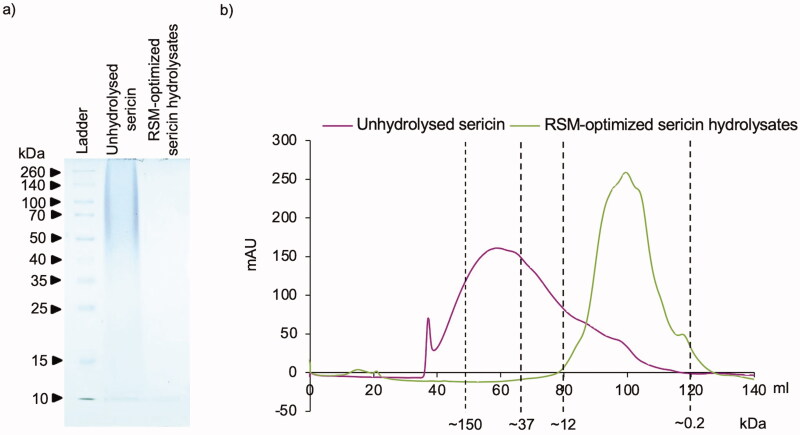
Molecular weight distribution of protein composition in unhydrolysed sericin and sericin hydrolysates prepared by using Alcalase^®^ under RSM-optimized condition in (a) SDS-PAGE analysis and (b) FPLC coupled with HiPrep 16/60 Sephacryl S-200 HR column.

The size distribution profile was also evaluated in sericin hydrolysates prepared under RSM-optimized condition via size exclusion chromatography using FLPC coupling with HiPrep 16/60 Sephacryl S-200 HR column. Like the distribution pattern observed in SDS-PAGE analysis (Figure 4a), the FPLC chromatogram illustrates that RSM-optimized sericin hydrolysates mainly contained with small protein (∼0.2–12 kDa) while the mixture of proteins ranging between 0.2 to higher than 150 kDa was presented in unmodified sericin (Figure 4(b)). These results suggest that greater antioxidant activity might result from the proteins at low molecular weight composing in RSM-optimized sericin hydrolysates.

## Discussion

Antioxidant peptides have been well recognized for their therapeutic potential and applicable benefits in diverse applications such as food additives and cosmeceutical ingredients (Wang et al. 2015; Jiang et al. 2017; Zhang et al. 2019). Recently, various uses of sericin protein present in the degumming water used in silk processing have been highlighted (Kunz et al. 2016; Cao and Zhang 2017; Liu et al. 2020). Silk sericin has potential for using in recycling industrial waste, but it is also potent for biological activity, which inspires the investigation of its antioxidant activity (Fan et al. 2010; Ersel et al. 2016; Ampawong et al. 2017; Manesa et al. 2020). It has been revealed that peptide characteristics, including molecular weight, amino acid sequence and hydrophobicity strongly determine its antioxidant potential (Karamać et al. 2016). Corresponding with the results presented in this study, enzymatic modification obviously alters the size distribution patterns (Figure 1) and radical scavenging activities of silk sericin protein (Table 3). Due to the possibility of specific scavenging activity being modulated by the definite features of peptide (Karamać et al. 2016), sericin hydrolysates obtained from trypsin and papain enzymatic reactions demonstrated lower ferric-reducing antioxidant power compared with both unhydrolysed sericin and sericin hydrolysates derived from Alcalase^®^ (Table 3). It should be noted that maximum antioxidant activity of protein hydrolysates requires suitable molecular distribution (Wu et al. 2008; Fan et al. 2010; Thongsook and Tiyaboonchai 2011). Sericin hydrolysates obtained from Alcalase^®^ reaction mostly composed with peptides at ∼10 kDa meanwhile larger and smaller peptides were respectively found in papain and trypsin sericin hydrolysates (Figure 1). The substrate specificity to aromatic amino acids as well as the capability to cleave both terminal and non-terminal peptide bonds might involve with the size distribution ranging between ∼10–100 kDa in sericin hydrolysates derived from papain (Berger and Schechter 1970). Despite being an endopeptidase, high containing of lysine and arginine, the specific substrates for trypsin, in silk sericin protein could result in smaller size of sericin hydrolysates modified by trypsin compared to sericin hydrolysates prepared by using Alcalase^®^ (Sprang et al. 1988).

Scavenging activity against free radicals, which is one of the important machineries of antioxidant compounds, can be achieved through the translocation of single electrons or hydrogen atoms to free radical molecules (Ndhlala et al. 2010; Apak et al. 2016). Therefore, the antioxidant capability of sericin hydrolysates obtained from three commercial enzymes was evaluated through DPPH and FRAP assays for determination of single electron donation, as well as ORAC assay for evaluating the translocation of hydrogen atoms herein. When compared with unmodified, trypsin- and papain-hydrolysed sericin, Alcalase^®^ sericin hydrolysates possessed the highest scavenging capacities against all three free radicals (Table 3). Alcalase^®^ is widely used for the enzymatic modification of various proteins for specific purposes because of its broad substrate specificity and commercial availability (Puangphet et al. 2015; da Silva et al. 2018; Kubglomsong et al. 2018). The obtained results presented in Table 3 concur with a previous study into the highest % inhibiting DPPH and ferric-reducing power of sericin hydrolysates derived from Alcalase^®^ compared with various protease enzymes (Fan et al. 2010). In contrast, the reduction of ferric-reducing power was indicated in trypsin- and papain-hydrolysed sericin. It is the fact that the less correlation with other antioxidant assays and the underestimating ROS scavenging activity of hydrogen-transferring molecules, especially antioxidant peptide, has been reported as the limitations of FRAP assay (Ou et al. 2002). Nevertheless, FRAP value is established to represent the capability of antioxidant to maintain cellular redox status and stop oxidative chain reaction in biological sample (Prior et al. 2005). Taken together with the greater ROS scavenging activity through hydrogen atom translocation assessed via ORAC assay, these data clearly suggest that sericin hydrolysates derived from Alcalase^®^ modification are a candidate for antioxidant peptides through mediating single electron and hydrogen atom transfer.

In order to maximize the antioxidant activity of Alcalase^®^ sericin hydrolysates, ROS scavenging activities of sericin hydrolysates released from Alcalase^®^ at various enzymatic conditions were simulated through RSM. Under optimized conditions of pH: 7.5, enzyme/substrate ratio: 1.5 (w/w) and temperature: 70 °C obtained from numerical optimization in Design-Expert^®^ 11 software, sericin hydrolysates demonstrated scavenging activities assessed through DPPH, FRAP and ORAC assays close to predicted values (Table 8). Response surface models are considered reliable when the response conducted under recommended optimum conditions contains % error from the model-predicted value lower than 5% (Mia and Dhar 2016; Mukhopadhyay et al. 2019). For % inhibition of DPPH, the low % error between actual and predicted response of sericin hydrolysates (Table 8) corresponded with the predicted R^2^ value obtained from multiple linear regression analysis (Table 6). The predicted R^2^, which is usually lower than R^2^ value is a statistical term presenting the suitability of using a regression model for prediction of a new observed response. Despite having the lowest predicted R^2^ value (0.4991) among the three regression response models, the greatest correlation between predicted and conducted responses of FRAP assay was obtained from Alcalase^®^ sericin hydrolysates. In contrast, the highest difference from the predicted response of sericin hydrolysates prepared by using Alcalase^®^ according to RSM conditions was observed in scavenging activity against ROO**˙** (Table 8). Variations in the ORAC response of sericin hydrolysates might result from the fact that only ROO**˙** scavenging activity can be significantly altered through the modification of all three variables, pH (A), enzyme/substrate ratio (B) and temperature (C), as evidenced by *p* being < 0.05 in linear (A, B and C) and quadratic (A^2^, B^2^ and C^2^) effects in Table 7.

Notably, the ROS scavenging activities of % DPPH inhibition, ferric-reducing power and oxygen radical absorbance capacity were comparable between sericin hydrolysates derived from optimized RSM (Table 8) and the manufacturers’ recommended conditions (Table 3). The adjustment of pH and/or temperature according to the active enzymatic conditions of Alcalase^®^ (pH 6.5-8.5, 60 °C) might be considered to minimize the production costs. Because substantial alteration of hydrolysed proteins is obtained after 3 h of Alcalase^®^ enzymatic reaction (Puangphet et al. 2015), antioxidant sericin hydrolysates should be achieved after at least 3 h of enzymatic modification. Additionally, the dramatically augmented antioxidant capacity of sericin hydrolysates in H_2_O_2_-treated human keratinocytes and melanocytes compared with a well-known antioxidant (NAC) and unmodified sericin (Figure 3) verify its biological activity. It is worth noting that the secondary structures, particularly β-sheet considerably contribute to free radical scavenging activity and cellular antioxidant potential of unmodified sericin solution though composing mainly of protein at high molecular weight (Figure 4) (Jandaruang et al. 2012; Lamboni et al. 2015; Yuan et al. 2018; Zhu et al. 2021). Indeed, the highest ratio of β-sheet structure was also revealed in the protein hydrolysates prepared by Alcalase^®^ compared with various enzymatic modifications (Zhu et al. 2021).

## Conclusions

The optimum enzymatic conditions for the preparation of sericin hydrolysates with high potency for scavenging activity against diverse free radicals and biological antioxidant activity were revealed in this study. The acquired RSM information would be benefit for developing antioxidant peptide from diverse resources, especially the recycling of waste products from silk industry.

## Data Availability

All data generated or analysed during this study are included in this article.
